# Transmitting biomolecular chirality into carbon nanodots: a facile approach to acquire chiral light emission at the nanoscale†

**DOI:** 10.1039/d2sc05794h

**Published:** 2022-11-28

**Authors:** Sonia Maniappan, Kumbam Lingeshwar Reddy, Jatish Kumar

**Affiliations:** a Department of Chemistry, Indian Institute of Science Education and Research (IISER) Tirupati Tirupati – 517507 India jatish@iisertirupati.ac.in

## Abstract

Since the observation of chirality at the nanoscale, research focused towards the design and synthesis of optically active nanomaterials has been at a brisk pace. In this regard, carbon based zero dimensional nanomaterials have attracted vast attention due to their rich optical properties, abundance of raw materials, minimal environmental hazardousness, good solubility, and ease of surface modification. However, efforts focused towards the synthesis of chiral carbon nanodots exhibiting optical activity both in their ground and excited states are rather scarce. Herein, we report a facile synthetic approach for the preparation of three sets of intrinsically chiral carbon nanodots that exhibit intense circularly polarized luminescence. Synthesis under optimized conditions using l- and d-isomers of the chiral precursors led to the formation of carbon nanodots that displayed mirror image circular dichroism and circularly polarized luminescence signals revealing their ground and excited state chirality. The experimental results are supportive of the reported core–shell model comprising an achiral carbon core that is enclosed within an amorphous shell contributing to the chiral luminescence. The luminescence anisotropy and wavelength could be tuned by varying the experimental conditions such as temperature and pH. The chiral emissive properties of the nanoparticles could be demonstrated in free-standing polymeric films revealing their potential to be used as chiral light emitting agents in optical devices, data storage and security tags. Being the first observation of intrinsic circularly polarized luminescence from a range of carbon nanodots, both in the solution and solid state, we envisage that the work will open new avenues for the investigation of excited stated chirality at the nanoscale.

## Abstract 2

A facile approach using simple biomolecules as precursors is adopted for the synthesis of three sets of optically active carbon nanodots that exhibited circularly polarized luminescence in both solution state and in solid films.

## Graphical abstract

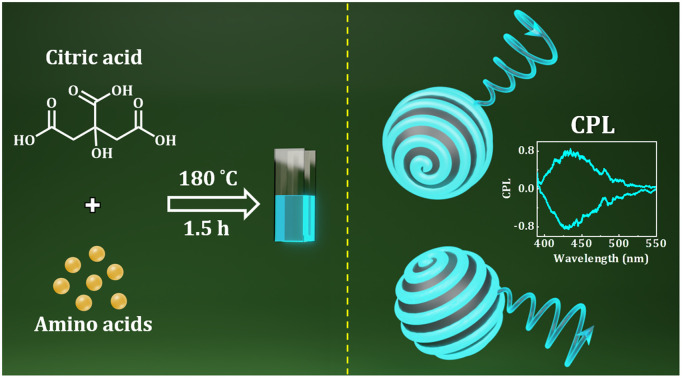

## Introduction

Chirality is a unique geometric property covering different hierarchical scales ranging from subatomic particles through molecules to galaxies.^1,2^ Captivated by symmetry principles, research has been focused towards mimicking the optical activity in natural objects to create chiral functional materials at different length scales.^3^ Of particular interest has been the observation of chirality in nanoparticles, both from its fundamental and application standpoints. Since its inception, the growth of nanoscale chirality has been at a brisk pace and optical activity is being unravelled in a variety of new materials.^4^ These include organic^5^ and inorganic nanostructures such as semiconductor quantum dots (QDs),^6^ perovskite nanocrystals,^7^ carbon nanomaterials,^8^ plasmonic materials,^9–11^ and metal or covalent organic frameworks and their assemblies.^12,13^ Among them carbonaceous materials have attracted vast interest due to their abundance, environmentally benign nature, low photobleaching, biocompatibility and the ability to form covalent bonds with different hybridization states.^14^ Herein, we focus our interest on the fabrication of a set of chiral carbon nanodots (CNDs), a zero-dimensional carbon-based nanomaterial typically of size less than 10 nm, exhibiting intense optical activity both in its ground and excited states.

**Fig. 1:**
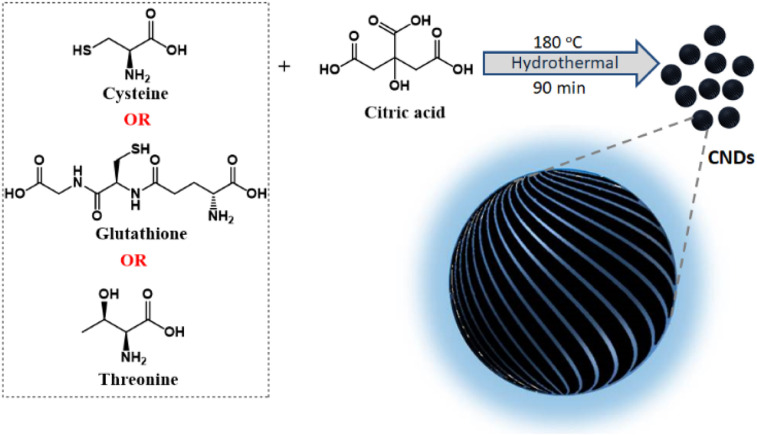
Scheme illustrating the synthesis of chiral CNDs using a combination of citric acid and different chiral agents as precursors.

Chirality in nanostructures can be achieved majorly through three different approaches; (i) generation of intrinsic chirality due to structural distortion, (ii) chiral induction through functionalization of ligands, and (iii) template-assisted chirality.^15–17^ While the latter approaches have been well explored, the fabrication of materials possessing intrinsic chirality has remained a challenge.^18^ Synthesis of inherently chiral materials is of extreme importance as deep knowledge on their optical activity could help unravel the mysteries related to the origin of homochirality in nature.^19^ Circular dichroism (CD) has been extensively used for the investigation of ground state chirality in molecules and materials.^20^ However, circularly polarized luminescence (CPL), the luminescence counterpart of CD, is gaining vast attention in recent years due to its potential to explore the excited state chirality.^21,22^ In this regard, CPL properties of various nanomaterials such as semiconductor QDs and perovskite nanocrystals have been investigated.^23^ The chiral emissive properties of CNDs have also been studied by exploiting template-assisted methods.^24^ Zheng *et al.* reported multicolour CPL using cellulose nanocrystals as templates^25^ whereas Ru *et al.* adopted a supramolecular approach using polymers to achieve CPL.^26^ We have recently demonstrated multicolour CPL from CNDs appended on chiral gel as templates.^27^ However, direct synthesis of intrinsically chiral emissive CNDs has remained a challenge and a few investigations in this direction have reported zero CPL for chiral CNDs.^28,29^ Hence, research focused towards intrinsically chiral CNDs and optimization of their synthesis strategies for enhanced excited state optical activity is new. Herein, we for the first time report the synthesis of different sets of CNDs that exhibit chiral light emission both in the solution and solid state. Adopting a facile synthetic approach, a general design strategy is developed for the synthesis of CPL active CNDs (Fig. 1).

## Results and discussion

### Structural characterization of carbon nanodots

**Fig. 2:**
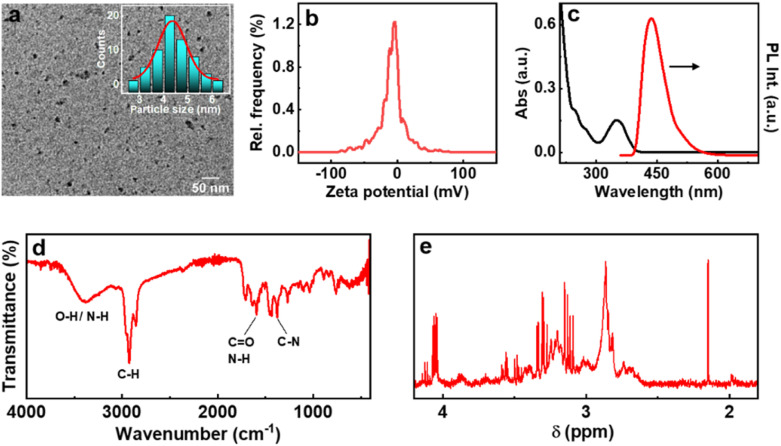
(a) TEM image, (b) zeta potential plot, (c) UV-visible absorption (black trace) and fluorescence (red trace) spectra, (d) FT-IR and (e) NMR spectra of Cys-CNDs. Inset in ‘a’ shows the histogram depicting the size distribution of the particles.

Among the various strategies reported for the synthesis of CNDs, solution-based hydrothermal method using organic small molecules has been identified as a versatile approach for the generation of high-quality nanoparticles.^30^ Herein, we use a combination of citric acid along with biologically important chiral molecules (cysteine, threonine and glutathione) as precursors for the synthesis of optically active CNDs (Cys-CNDs, Thr-CNDs and Glu-CNDs) (Fig. 1). Citric acid functions as the carbon source that undergoes carbonization at higher temperature whereas chiral biomolecules act as the chirality inducing agent. The synthesized CNDs were purified by adopting techniques like precipitation and dialysis (see ESI†). The morphological characteristics of the purified CNDs were studied using high-resolution transmission microscopy (HRTEM). The tiny dark spots present in the TEM micrographs indicated the formation of Cys-CNDs possessing an average size of 4.3 nm (Fig. 2a). The average size was measured to be 2.4 nm and 2.3 nm for the Thr-CNDs and Glu-CNDs respectively (Fig. S1†). Zeta potential measurements revealed that the particles carried a negative surface charge between −4.4 and −10.3 mV for the three sets of CNDs (Fig. 2b and S2†). Fourier transform infrared (FT-IR) spectroscopy was performed to validate the composition, surface group and bonding type in the synthesized CNDs. The FT-IR spectrum for all three CNDs showed the presence of multiple functional groups corresponding to the precursors used in the synthesis (Fig. 2d and S3†). A broad and intense band at 3100–3500 cm^−1^ is attributed to the H-bonding of the O–H and N–H groups. Peaks observed in the region of 2850–2950 cm^−1^ are typical for the sp^3^ C–H stretching vibration. A prominent peak at ∼1700 cm^−1^ in all the three sets of CNDs is attributed to the C=O stretching of amidic functional groups formed during the hydrothermal synthesis. This peak is more intense for Thr-CNDs, indicating the presence of more C=O groups on the surface. Peaks at 1580 and 1620 cm^−1^ can be attributed to the N–H bending and C=O stretching of the amide groups, respectively. A peak at 1450 cm^−1^ associated with C–N stretching is more pronounced for all the samples. Peaks observed in the region 900–1200 cm^−1^ are attributed to the C–O, C–N, and C–H bonds which are more intense for Cys-CND and Glu-CND samples. A small peak at 1159 cm^−1^ could be attributed to the C–S bond (Fig. S4†). The typical S–H stretching peak at 2550 cm^−1^ was not seen indicating the absence of the free thiol group which might involve in the bond formation with carbon/nitrogen. The structural features of the CNDs were further investigated using NMR spectroscopy. Broad peaks in the aliphatic region (4.2–1.8 ppm) indicate that the hydrogen atoms experience a different environment and slow rotation in the Cys-CNDs (Fig. 2e).^31^ Similar plots were observed for the Thr-CNDs and Glu-CNDs confirming that the observed properties are characteristic of the particles (Fig. S5†). Diffusion-ordered spectroscopy (DOSY) has been employed as a powerful tool to establish the size of such nanoparticles. DOSY NMR spectra reveal that the CNDs are effectively larger in size than their corresponding precursors (Fig. S6 and ESI†).^32,33^ The calculated molecular weights for the three sets of CNDs are in the range between 648 and 1017 Da (Table S1†), in agreement with earlier reports on similar CNDs,^32^ and consistent with extended carbon nanostructure formation.

### Optical properties of chiral CNDs

The optical properties of the synthesized CNDs are largely dependent on the nature of the precursors used and the experimental conditions employed. The absorption spectra of Cys-CNDs synthesised through a reaction at 180 °C for 90 min revealed two distinct peaks at 246 and 351 nm (Fig. 2c). The peak at 246 nm corresponds to the π–π* transitions of the sp^2^ hybridised aromatic domain whereas the band at 351 nm could be ascribed to the n–π* transitions of the carboxyl group as well as the C=N/C=S bonds of the aromatic CND core.^34^ Analogous spectral profiles were observed for the CNDs synthesized using threonine and glutathione (Fig. S7a†). The synthesis generally involves a series of condensation reactions to generate fluorophores, which on further polymerization and carbonization yield CNDs.^35^ The luminescence properties of CNDs based on citric acid can be ascribed to the carbonaceous core or complex compositions of the carbon cores and organic fluorophores.^14^ Strong emission peaks were observed at 432, 425 and 430 nm upon excitation at 350, 325 and 328 nm for the Cys-, Thr- and Glu-CNDs respectively (Fig. 2c and S7b†). All three CNDs exhibited an excitation-independent emission pattern due to the presence of the same emissive state (Fig. S8†).^36,37^ The Cys-, Thr- and Glu-CNDs exhibited a reasonably high luminescence quantum yield of 17.25%, 8.65% and 16.74% respectively. The fluorescence lifetime plots displayed a biexponential decay with an average lifetime of 10.6, 12.5 and 10.5 ns for the major component in Cys-, Thr- and Glu-CNDs respectively, in agreement with the reported values (Fig. S9 and Table S2†).^38^

**Fig. 3:**
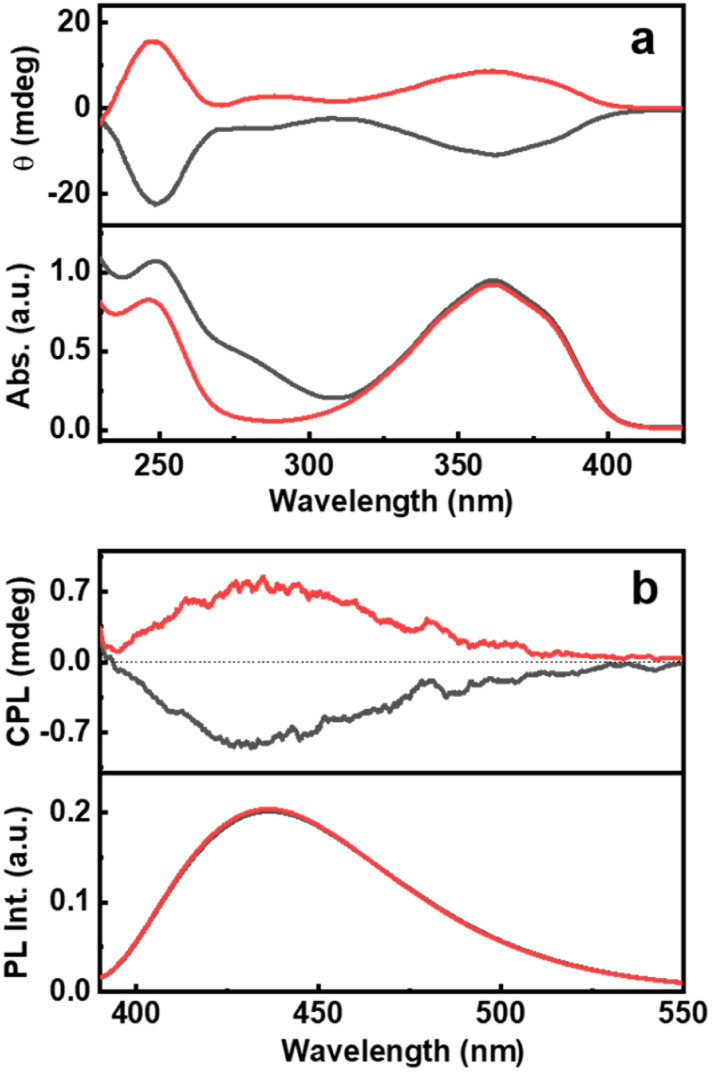
(a) CD and the corresponding absorption spectra and (b) CPL and the corresponding emission spectra of CNDs synthesised using l-cysteine (black traces) and d-cysteine (red traces) in the presence of citric acid.

The ground state chirality of the CNDs was analysed using CD spectroscopy. Mirror image CD signals with positive sign for the d-Cys-CNDs and negative sign for the l-Cys-CNDs confirmed the formation of chiral nanoparticles. The CD spectrum displayed two peaks at 246 and 351 nm in accordance with the absorption profile (Fig. 3a). The spectral features are ascribed to the chirality induced through the hybridization of chiral precursors with the CNDs. Moreover, the CD signals are well separated from the optical signatures of the cysteine molecule ruling out the direct contribution of the reaction precursors.^39^ The quantification of chiral signals was done by evaluating the dissymmetry factor (*g*_abs_). All three sets of CNDs exhibited *g*_abs_ values in the range of 10^−4^ (Table S3†). The major focus of our investigations was to study the excited state chiral properties of the synthesised CNDs using CPL spectroscopy. Interestingly, clear mirror image CPL profiles with positive sign for d-Cys-CNDs and negative sign for l-Cys-CNDs were observed upon excitation at 350 nm (Fig. 3b). The CPL peak maxima were centred around 432 nm, the emission wavelength of the corresponding CNDs. While there are reports on the ground state chiral investigations of such particles,^39,40^ to the best of our knowledge this is the first observation of CPL from CNDs synthesized using simple amino acids as chiral agents. The extent of chiral dissymmetry is quantified using the anisotropic factor (*g*_lum_) which is given by *g*_lum_ = 2(*I*_L_ − *I*_R_)/(*I*_L_ + *I*_R_), where *I*_L_ and *I*_R_ are the intensities of the left- and right-circularly polarized light, respectively.^41^ The CNDs exhibited a *g*_lum_ value of 8 × 10^−4^ and −7 × 10^−4^ at 432 nm for the particles synthesized using d- and l-cysteine respectively. The observed *g*_lum_ is well within the range of what has been reported for organic molecules or other chiral nanosystems. The CPL and CD peaks exhibit similar sign and anisotropy values confirming the retention of the chiral orientation of nanoparticles in their ground and excited states.

The origin of fluorescence in CNDs has been a matter of debate.^42^ The fluorescence in CNDs synthesized using citric acid and cysteine has been reported to be due to the formation of intermediates like 5-oxo-3,5-dihydro-2*H*-thiazolo[3,2-*a*]pyridine-3,7-dicarboxylic acid (TPDCA) that either undergoes polymerization and carbonization or attaches to the carbon core to form the CNDs.^43–45^ To verify the contribution of TPDCA to the chiral anisotropy of CNDs, TPDCA was independently synthesized and characterised (see ESI†). While TPDCA exhibited luminescence, it did not show any chiral signals ruling out its role in the observed optical activity of the CNDs (Fig. S10†). To further investigate the role of citric acid and chiral precursors, synthesis was performed by heating the reactants separately at 180 °C. The luminescence of the resulting samples was extremely weak compared to that of the CNDs synthesised under the same conditions with a combination of citric acid and amino acid (Fig. S11†). The chiral signals were also absent in these samples highlighting the role of multiple reagents in the synthesis protocol (Fig. S12†). Hence, the chiral nature must be induced by the chiral precursors along with the carbon source (citric acid) that undergoes reaction and forms a part of the CND surface (*vide infra*).

**Fig. 4:**
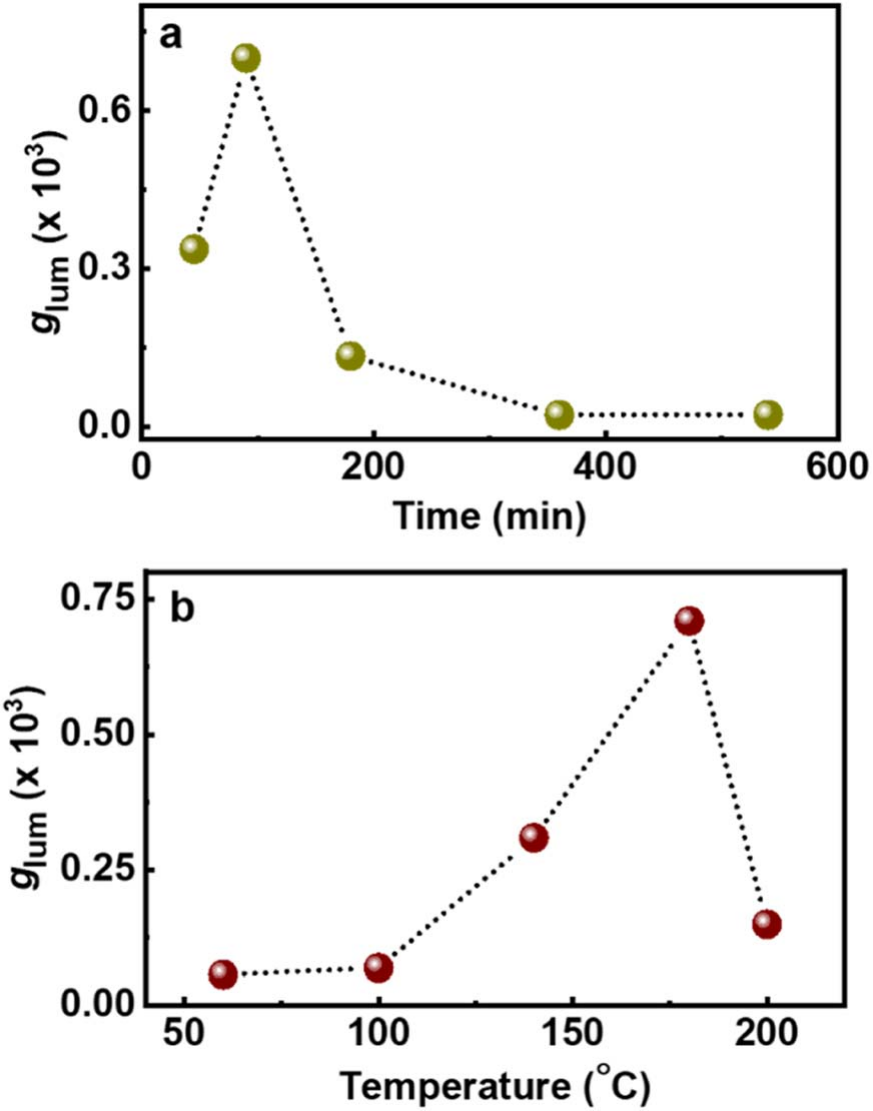
Plots depicting the changes in *g*_lum_ values upon changing the (a) reaction time and (b) reaction temperature during the synthesis of chiral light emitting Cys-CNDs.

In addition to the role of reaction precursors, the optical activity is strongly dependent on synthetic conditions applied. Hence, to study the role of experimental parameters, and to obtain enhanced luminescence anisotropy from the particles, we tried to vary the reaction conditions. To investigate the correlation of reaction time with the chiroptical properties, CNDs were synthesized by varying the duration of the reaction (45, 90, 180, 360 and 540 min). An initial increase in the CPL intensity was observed up to 90 min, which diminished with a further increase in reaction time (Fig. 4a). These observations were in corroboration with the CD spectral changes indicating that a reaction time of 90 min is optimal for the formation of a well hybridized carbon core supported by the surface ligands (Fig. S13†).^46^ On further increasing the reaction time, the carbonization of the surface occurs along with the breakdown of chiral derivatives present on the surface which in turn leads to the reduction in optical activity. Similar investigations on the effect of reaction temperature showed a gradual increase in CPL intensity that saturated at 180 °C followed by a decrease at further higher temperatures (Fig. 4b and S14†). CD spectra also followed a similar trend confirming that the chiral surface is prominent at an optimal reaction temperature of 180 °C. The temperature and time-dependent studies are an indirect proof for the reported core–shell model of CNDs that results in chiral light emission (*vide infra*). Under optimal reaction conditions, the nanoparticles form a carbonaceous core that is surrounded by a luminescent chiral shell. With increasing reaction time (or temperature), a gradual decrease in the intensity of the CPL signal is observed and this can be attributed to a higher degree of carbonization that break up the chiral structure of the ligands incorporating into the central carbon core.^8^

**Fig. 5:**
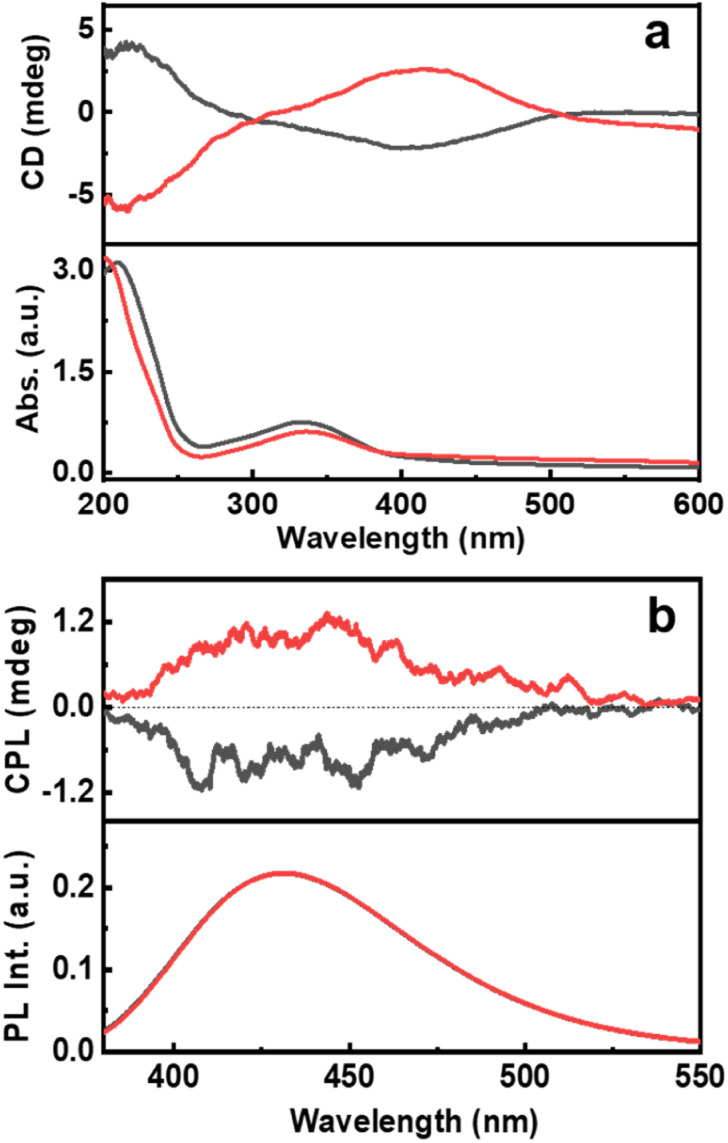
(a) CD and the corresponding absorption spectra and (b) CPL and the corresponding emission spectra of CNDs synthesised using l-threonine (black traces) and d-threonine (red traces) in the presence of citric acid.

For any new observation, it is important that the phenomena be demonstrated in multiple systems to establish the generality of the technique. To probe the chiral emissive properties in different CNDs, CPL investigations were carried out on CNDs synthesized using citric acid as the common precursor along with threonine and glutathione as the chiral agents. Investigations of the chiroptical properties revealed mirror image CD profiles at the corresponding absorption wavelengths for the CNDs synthesized using the two isomers of threonine (Fig. 5a). Interestingly, intense CPL signals with positive and negative signs for the CNDs synthesized using d- and l-threonine were observed at the corresponding luminescence wavelengths (Fig. 5b). The sign of CD and CPL plots was in good agreement. The *g*_lum_ values were found to be −2.1 × 10^−4^ and 2.6 × 10^−4^ for l and d isomers respectively. Similar observation with CNDs synthesized using glutathione could establish the generality of the technique; CD and CPL plots at the corresponding UV-vis and fluorescence wavelengths were observed for the CNDs synthesized using l-glutathione (Fig. S15†). Due to the non-availability of opposite isomers, we could not obtain mirror image CD and CPL plots for Glu-CNDs. The nature of CPL signals was similar in all three sets of CNDs, however, the peak position and the *g*_lum_ values varied slightly depending on the nature of CNDs formed using the different chiral precursors (Table S4†). Apart from these three chiral precursors discussed, efforts were also made to synthesize CNDs using other amino acids like lysine, serine, methionine and cystine. Even though the CNDs synthesised from these amino acids manifest high luminescence properties (Fig. S16†), they lack excited state chirality as confirmed by the absence of CPL peaks in the fluorescence region (Fig. S17†). Hence, the analysis of various amino acids revealed that the structural parameters are crucial to the formation of CPL active CNDs. The strong interaction of amino acids (chiral precursor) with citric acid can result in carbonization, however, the optical activity of CNDs are due to the retention of the chiral character in nanoparticles, failing of which can lead to the formation of achiral luminescent CNDs.

Further investigations were carried out to tune the CPL signals. To achieve this, the synthesized Cys-CNDs were subjected to varying temperature and pH. The temperature dependent CPL studies showed that the absorption and CD remained unchanged upon heating the Cys-CNDs to 90 °C (Fig. S18a†). In contrast, a quenching of emission was observed on increasing the temperature and this in turn was reflected in the lowering of CPL signals (Fig. S18b†). The quenching in emission is attributed to the increase in the radiationless dissipative channels at higher temperature.^47,48^ The temperature dependence of emission was reversible upon cooling the sample back to 20 °C. The thermoresponsive nature of CPL and fluorescence of Cys-CNDs make them an excellent candidate for various sensing applications. The influence of pH on the stability and optical properties was also investigated. A significant red shift in CD and absorption bands was observed at lower pH (pH = 1). A similar bathochromic shift could be achieved in CPL and luminescence profiles accompanied by a slight quenching (Fig. S19†). The observed spectral changes can be attributed to the aggregation caused by strong intermolecular hydrogen bonding between the carboxyl and hydroxyl groups present in the CNDs.^49,50^ The absorption and emission characteristics at higher pH (till pH 11) suggest that the CNDs are stable over a broad pH range.^34^ Hence, changes in external parameters could establish the stability of chiral CNDs under varying conditions. Moreover, the position and intensity of the CPL signals could be successfully tuned to some extent.

**Fig. 6:**
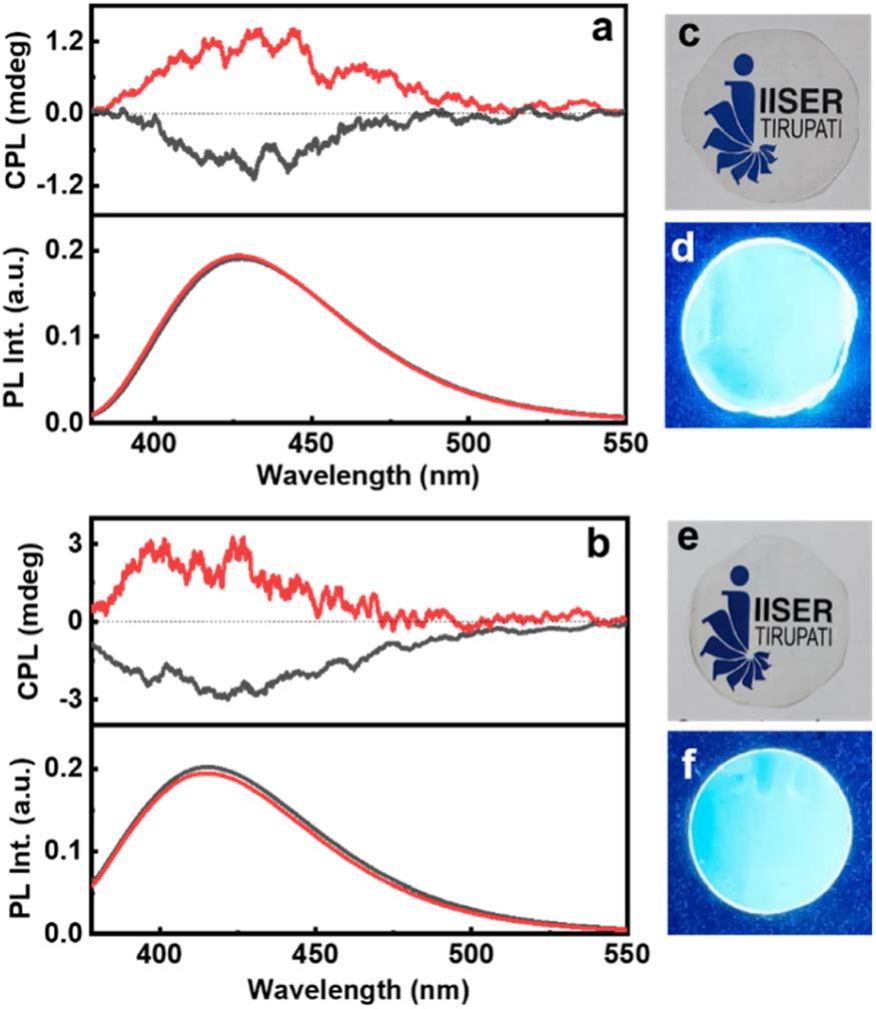
CPL and the fluorescence spectra of polymeric films of (a) Cys-CNDs and (b) Thr-CNDs. Black and red traces depict the spectra from the CNDs synthesized using l- and d-isomers respectively. Photographs of the PVA films synthesized by incorporating (c and d) Cys-CNDs and (e and f) Thr-CNDs captured under (c and e) day light and (d and f) UV light.

For any practical application, it is highly relevant that the properties observed in solution be replicated on solid surfaces. With an aim to demonstrate the optical characteristics of chiral CNDs in the solid state, we have fabricated self-standing transparent films of CNDs in 5% PVA (Fig. 6c–f). The CD spectra of the CNDs exhibited a mirror image profile for the nanoparticles synthesized using the opposite isomers of cysteine and threonine (Fig. S20†). Interestingly, the CPL investigations of the films revealed that the excited state chiral properties of the CNDs were retained in the solid state. Both Cys-CNDs and Thr-CNDs exhibited mirror image CPL plots for the nanoparticles prepared using the two isomers (Fig. 6). Similar effects were observed from CNDs synthesized using citric acid and glutathione confirming the repeatability of the process (Fig. S21†). The *g*_lum_ values as well as the sign of CPL in the films were in agreement with the observations in the solution state (Table S5†). The sign and *g*_lum_ values were consistent for CPL collected by rotating the film at different angles ruling out the possibility of any artefacts due to linear polarization effects. These results clearly indicate the retention of the chiral properties in the solid films. These results further emphasize the efficiency of these materials to function as effective CPL active systems in the solid state thereby revealing their capability as chiral light emitting materials that can find potential application in circularly polarized LEDs.

**Fig. 7:**
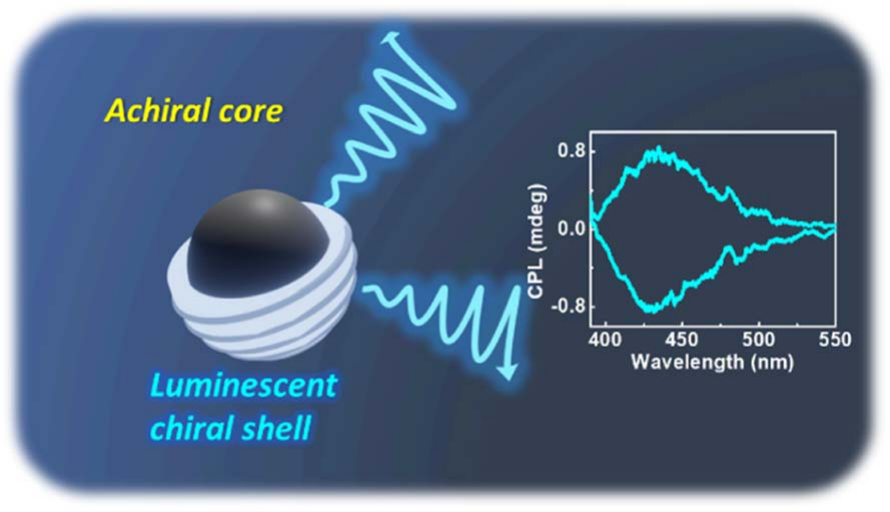
Scheme illustrating the mechanism of chiral light emission in optically active CNDs.

Based on the above results and discussions, the reported core–shell model of CNDs could be adopted to explain chirality evolution in these materials. As per the prescribed model, CNDs are composed of an achiral carbon core that is enclosed within an amorphous shell comprising fluorophores and conjugated structures which have abundant heteroatoms (Fig. 7). The chiral nature of the structures could be attributed to the shell comprising undecomposed amino acids or their derivatives connected by amide bonds.^51^ The hybridization of the electronic structure between the chiral residue in the shell and the achiral fluorophore units could lead to the generation of chiral hybridised states in the CNDs.^52^ Variations in the reaction conditions would primarily affect the shell and thereby the optical activity of the nanosystems. The hypothesis is supported by the loss of chirality upon increased reaction time/temperature during the synthesis of CNDs. Reduction of optical activity with the increase in reaction temperature (or time) suggests that the chiral derivatives undergo a higher degree of carbonization by breaking the chiral structure and subsequently incorporating into the central carbon core. The results prove that the selection of precursors and optimization of reaction conditions are crucial to the formation of a carbon core supported by a chiral luminescent surface that exhibits efficient luminescence anisotropy. Hence, the studies pave a new pathway for the development of a carbon-based nanosystem to accomplish efficient chiral light emission.

## Conclusions

In summary, a facile strategy was adopted for the synthesis of three sets of optically active CNDs. Reaction of the chiral precursors with citric acid under optimized conditions resulted in the formation of Cys-CNDs, Thr-CNDs and Glu-CNDs that exhibited chirality both in the ground and excited states. The chiral emission was demonstrated both in the solution state and in solid polymeric films of the nanoparticles. The evolution of chirality with respect to the reaction time and temperature was investigated to validate the core–shell structure composed of the carbon core and chiral shell. The CPL activity of CNDs could be tuned as a function of temperature and pH revealing the potential of the material in sensory applications. The demonstration of chiral light emission in CNDs coupled with their various other advantages, such as abundance of raw materials, ease of synthesis, good solubility, low toxicity, ease of surface modification, and resistance to photobleaching, makes these materials excellent candidates for application in display devices, optical data storage, security tags and biosensing.

## Data availability

All experimental details are added to the ESI file.†

## Author contributions

S. M. and K. L. R. contributed equally to the work. J. K. conceived and coordinated the project. S. M. and K. L. R. carried out the experiments. All authors analysed the data. S. M. and J. K. prepared the manuscript. All authors have given approval to the final version of the manuscript.

## Conflicts of interest

The authors declare no conflict of interest.

## Supplementary Material

SC-014-D2SC05794H-s001
