# Supplementary material for: Transmitting biomolecular chirality into carbon nanodots: a facile approach to acquire chiral light emission at the nanoscale
Source: Chem Sci. 2022 Nov 28;14(3):491–8. doi: 10.1039/d2sc05794h (PMC9847681; doi:10.1039/d2sc05794h)
Supplement: SC-014-D2SC05794H-s001 [file SC-014-D2SC05794H-s001.pdf]

Electronic Supplementary Material (ESI) for Chemical Science.
This journal is © The Royal Society of Chemistry 2022

Supporting Information

Transmitting Biomolecular Chirality into Carbon Nanodots: A Facile Approach to Acquire Chiral Light Emission at the Nanoscale

Sonia Maniappan, Kumbam Lingeshwar Reddy, Jatish Kumar*

Department of Chemistry, Indian Institute of Science Education and Research (IISER) Tirupati, Tirupati - 517507, India.
Email: jatish@iisertirupati.ac.in

Page No.	Contents
S3-S4	Experimental Procedures
S5	Fig. S1: TEM images and size distribution curve of Thr-CND and Glu-CND
S5	Fig. S2**:** Zeta potential curves of Thr-CNDs and Glu-CNDs.
S6	Fig. S3**:** IR spectra of Thr-CNDs and Glu-CNDs.
S6	Fig. S4: IR spectra depicting the comparison of synthesized CNDs and their corresponding precursors.
S7	Fig. S5: ^{1}H NMR spectra of Thr-CNDs and Glu-CNDs.
S7	DOSY NMR calculation for Cys-, Thr- and Glu-CNDs.
S9	Fig. S6: DOSY NMR plots for Cys-, Thr- and Glu-CNDs.
S10	Table S1: Molecular weight data of the CNDs
S10	Fig. S7**:** UV-vis and emission spectra of Thr-CNDs and Glu-CNDs.
S11	Fig. S8**:** Emission spectra of CNDs at different excitation wavelengths.
S11	Fig. S9**:** Lifetime decay curves of chiral CNDs.
S12	Table S2**:** Summary of the fluorescence lifetime data of CNDs.
S12	Table S3**:** g_{abs} values of the chiral CNDs.
S13	Fig. S10**:** UV-vis and CD profile of TPDCA molecule.
S13	Fig. S11**:** Emission spectra of precursors heated at 180°C
S14	Fig. S12**:** UV-vis and CD spectra of precursors heated at 180°C
S14	Fig. S13: Spectral properties of Cys-CNDs synthesized at different reaction time.
S15	Fig. S14**:** Properties of Cys-CNDs synthesised at different reaction temperatures.

S15 Fig. S15**:** Optical properties of Glu-CNDs in solution state.

S16 Table S4: g_{lum} values from chiral CNDs in solution state.

S16 Fig. S16**:** Excitation-dependent emission of CNDs with different amino acids.

S17 Fig. S17**:** Chirooptical spectra of CNDs synthesized using different amino acids.

S17 Fig. S18**:** Temperature dependent plots for Cys-CNDs.

S18 Fig. S19**:** pH dependent plots for Cys-CNDs.

S18 Fig. S20**:** Optical properties of CNDs in solid state.

S19 Fig. S21: Solid state CPL and emission spectra of Glu-CNDs.

S19 Table S5: g_{lum} values from chiral CNDs in solid state.

S19 References

Supporting Information

Experimental Procedures

Materials

L and D-cysteine monohydrochloride, L and D-threonine, L-glutathione reduced form, polyvinyl alcohol (PVA) are purchased from TCI India. Citric acid was obtained from Merck. All reagents and chemicals were used as received without any further purification. Milli-Q water was used for all the synthesis and other experiments.

Synthesis

Synthesis of blue emitting chiral CNDs

Equal weight (0.3 g) of citric acid and L-cysteine was dissolved in 6 mL of distilled water and mixed for 10-15 minutes. Then the whole solution mixture was transferred into Teflon lined autoclave to heat at 180 ^{o}C for 45, 90, 180, 360 and 540 minutes. After the reaction, the container was allowed to cool to room temperature in normal conditions. The reaction mixture was dried using rota evapouaration and the resulting gel like material was washed five times with 20 mL DCM. The DCM solution was added, sonicated and decanted each time to remove the organic impurities. After multiple washing with DCM and drying, the precipitate was dispersed in water and subjected to syringe filtration (0.22 µm syringe filter) and then kept for dialysis in cellulose tubing membrane having molecular weight cut off between 12,000 to 14,000 Da for 5 days.
Similar strategy was adopted for the synthesis of D-Cys CNDs, L & D-Thr CNDs and L-Glu CNDs using D-cysteine, L & D-threonine and L-glutathione respectively as chiral precursors. Glu-CNDs were purified using silica based column chromatography. Chiral luminescent fraction was collected by using a mixture of dichloromethane and methanol (90:10 *v/v*) solvents as an eluent. Thr-CNDs were purified through dialysis procedure for 2 days. All the obtained CNDs were stored in light free conditions.
The yield was calculated to be 24.3% (w/w), 18.5% (w/w) and 9.98% (w/w) for the Cys-, Thr- and the Glu-CNDs, respectively.

Synthesis of TPDCA

0.01 mol of citric acid and same molar ratio of L-cysteine was weighed and finely crushed with a mortar pestle. The solid mixture was then transferred into a beaker and heated at 150 ^{o}C for 1.5 h. The yellow gel-like material obtained after the synthesis was dissolved in deionized water. The product was then suction filtered and washed multiple times with cold water. The resulting precipitate was dissolved in 20 mL of hot water (90 ^{o}C) for recrystallisation. The crystals were obtained after 24 h. The final product was characterised

by NMR spectroscopy. ^{1}H NMR (400 MHz, DMSO-d_6) δ 6.58 (d, J = 19.3 Hz, 1H), 5.47 (d, J = 8.8 Hz, 1H), 3.91 (dd, J = 12.0, 9.0 Hz, 1H), 3.61 (d, J = 12.0 Hz, 1H).

Preparation of solid films

In a typical experiment, 5 wt% aqueous solution of PVA was prepared. The solution was then heated in water bath at 80 ^{o}C and was allowed to cool to ambient temperature. After cooling, 500 µL of CNDs were added to the solution and kept under vigorous stirring for 1.0 h. The solution was poured onto polystyrene Petri dishes (3.5 mm) and allowed to dry for 4 days under ambient conditions giving rise to free-standing film.

Characterization

The UV-vis characterization was carried out on Agilent Cary-3500 UV-vis spectrophotometer. The fluorescence spectra were measured on JASCO-8500 fluorescence spectrophotometer. CD spectra were recorded on JASCO-1500 CD spectrometer and CPL was measured using JASCO CPL 300 spectrophotometer. All the measurements were performed on 10 mm path-length quartz cuvette at room temperature. The lifetime measurements were performed on Edinburg FLS-1000 fluorescence spectrometer exciting the sample at 375 nm with a picosecond pulsed diode laser (EPL-375 Edinburgh Instruments). Absolute quantum yield measurements were carried out using the integrating sphere in an Edinburg FLS-1000 instrument. Zeta potential measurements done on Anton Paar Litesizer 500. The FT-IR spectra were recorded on PerkinElmer Spectrum Two FT-IR spectrometer. The ^{1}H NMR and DOSY NMR spectra of the synthesized CNDs were recorded on Bruker Avance Neo 400 spectrometer (400 MHz) after dissolving the samples in D_2O. The morphological characteristics of CNDs were acquired using Transmission electron microscopy (TEM) images using Jeol/JEM 2100 at 200 kV.

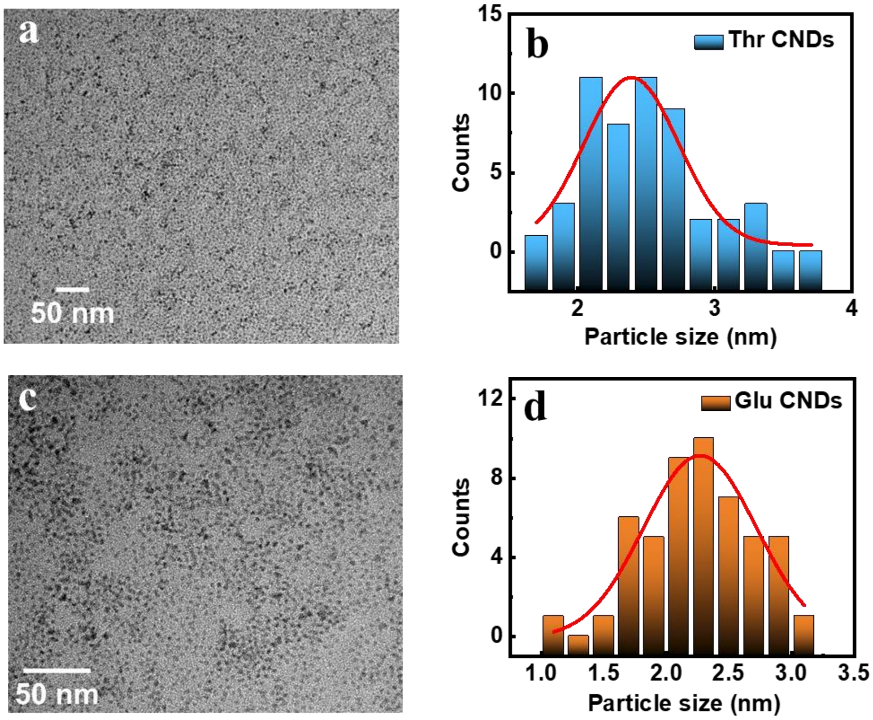

Figure S1. (a,c) TEM images and (b,d) size distribution plots of CNDs synthesized using (a,b) citric acid and threonine (Thr-CNDs) and (c,d) citric acid and glutathione (Glu-CNDs).

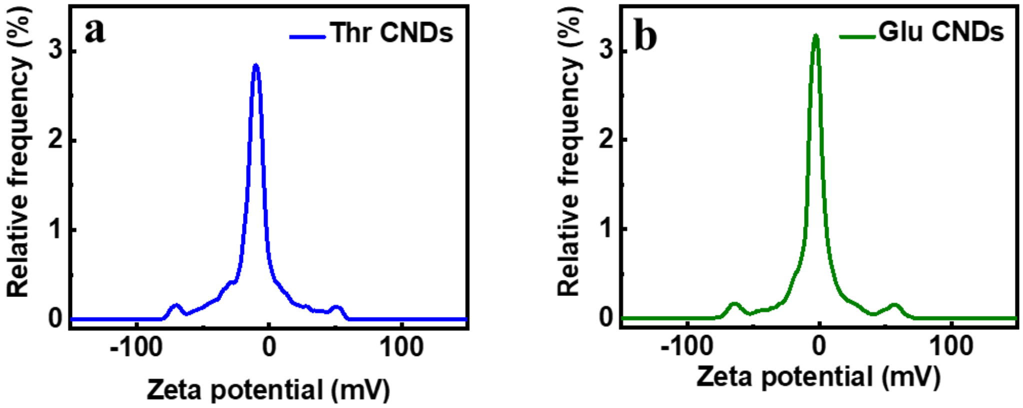

Figure S2. Zeta potential plots of (a) Thr-CNDs and (b) Glu-CNDs.

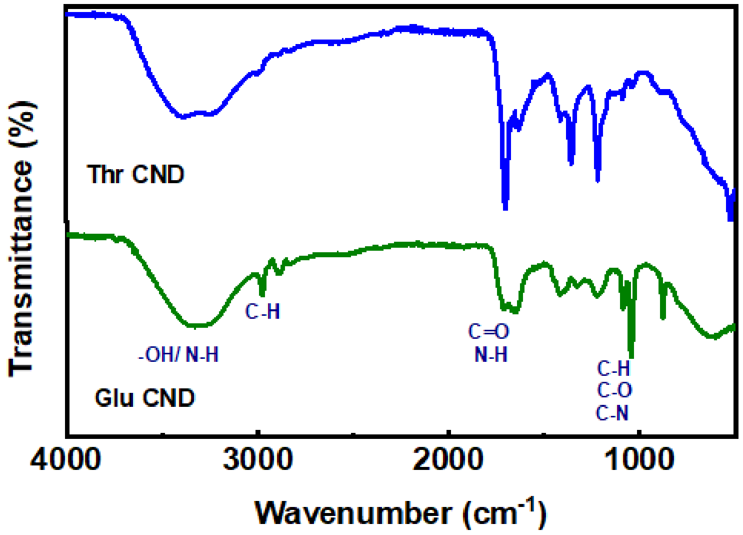

Figure S3. FTIR spectra of Thr-CNDs (blue trace) and Glu-CNDs (green trace).

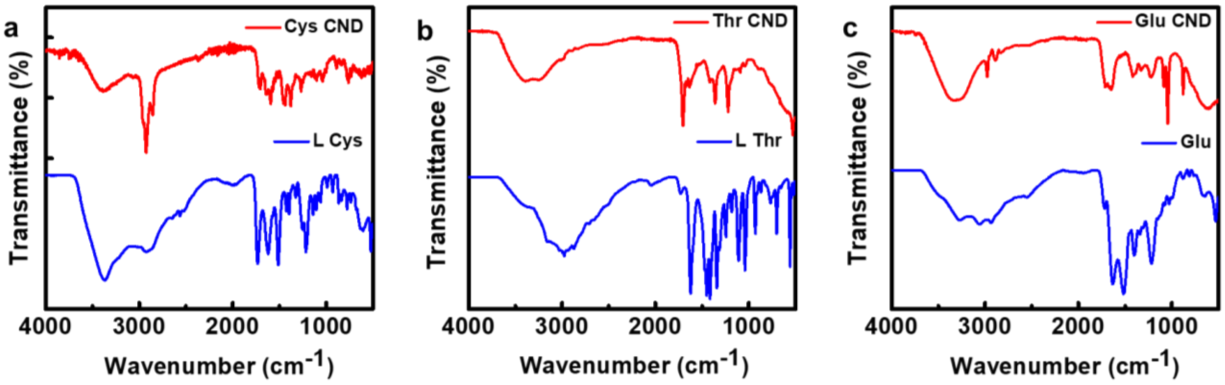

Figure S4. IR spectra depicting the comparison of synthesized CNDs (red traces) and their corresponding chiral precursors (blue traces) for (a) Cys-CNDs, (b) Thr-CNDs, and (c) Glu-CNDs.

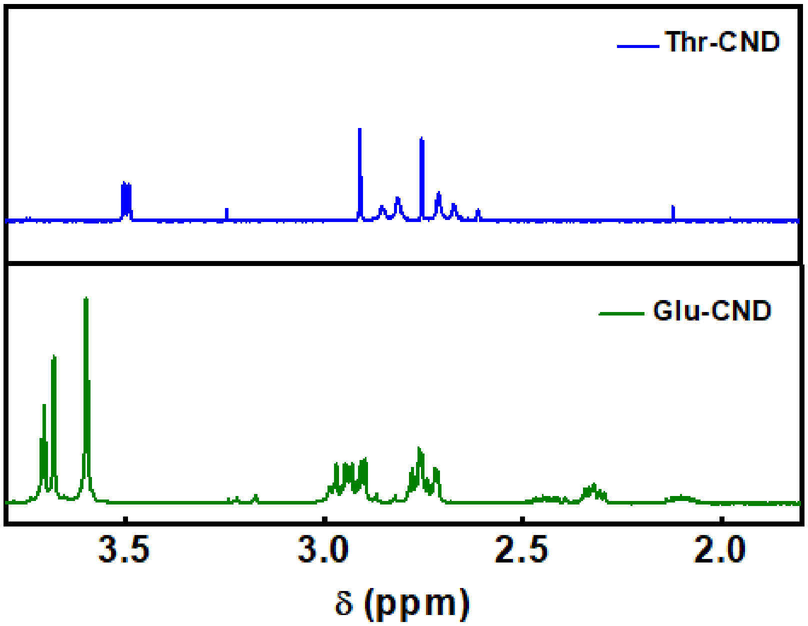

Figure S5. ^{1}H NMR spectra of Thr-CNDs (blue trace) and Glu-CNDs (green trace).

DOSY NMR analysis

The DOSY NMR was collected to study the nature of CNDs.[1,2] Assuming spherical structures for the CNDs, diffusion coefficient (Δ) was correlated to its hydrodynamic radius using Stokes-Einstein equation as given below

$$\Delta = \frac{k_B * T}{6\pi\eta r_H}$$

On comparing the Δ values, normalized to the solvent, CNDs and a known reference, the ratio of the diffusion coefficient can be compared to the ratio of the radius and can be further compared to the volume of particles.

$$\frac{\Delta_C}{\Delta} = \frac{r}{r_c}$$

$$\left(\frac{\Delta_C}{\Delta}\right)^3 = \frac{V}{V_C}$$

On applying Graham's law, diffusion coefficient can be connected to the mass of the compound taken for the analysis

$$\Delta = k * \left(\frac{T}{m}\right)^{1/2}$$

The constant k depends on different geometric factors which can affect the diffusion of the particles in solution. By the assumption that k is same for two compounds, the relative diffusion coefficient of carbon nanodots (Δ) and the precursor (Δc) is given by

$$\left(\frac{\Delta_C}{\Delta}\right)^2 = \frac{m}{m_c}$$

Therefore, the approximate mass of the carbondots (m) can be calculated to be

$$m = m_c \left(\frac{\Delta_C}{\Delta}\right)^2$$

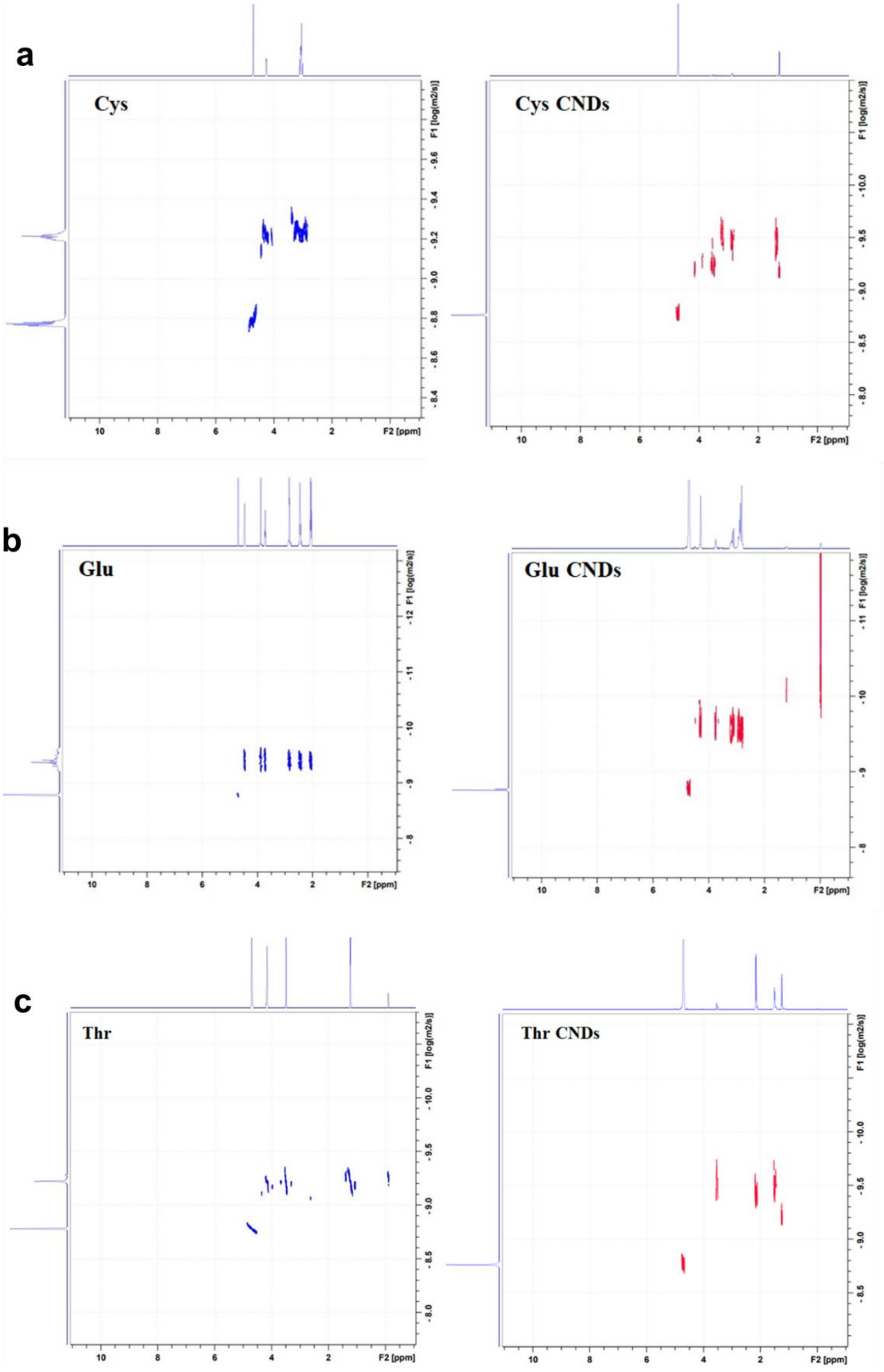

Figure S6. 2D DOSY NMR plots of purified (a) cysteine (blue trace) and Cys-CNDs (red trace), (b) threonine (blue trace) and Thr-CNDs (red trace), and (c) glutathione (blue trace) and Glu-CNDs (red trace).

Table S1. Calculated parameters using DOSY NMR for CND samples.

CNDs	R_{rel}	V_{rel}	Estimated Molecular weight (Da)
Cys-CNDs	2.22	11.037	870.6
Thr-CNDs	2.33	12.68	647.8
Glu-CNDs	1.82	6.0	1017.9

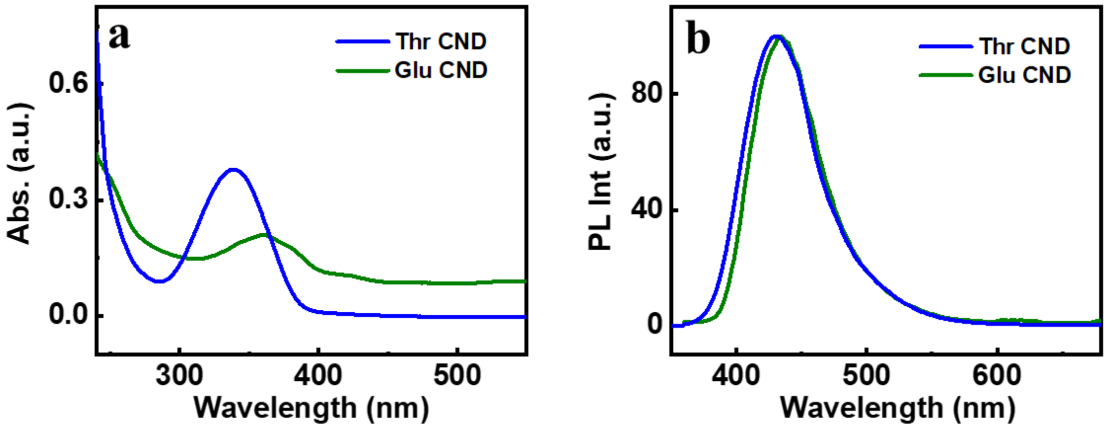

Figure S7. (a) UV-vis and (b) fluorescence spectra of Thr-CNDs (blue traces) and Glu-CNDs (green traces).

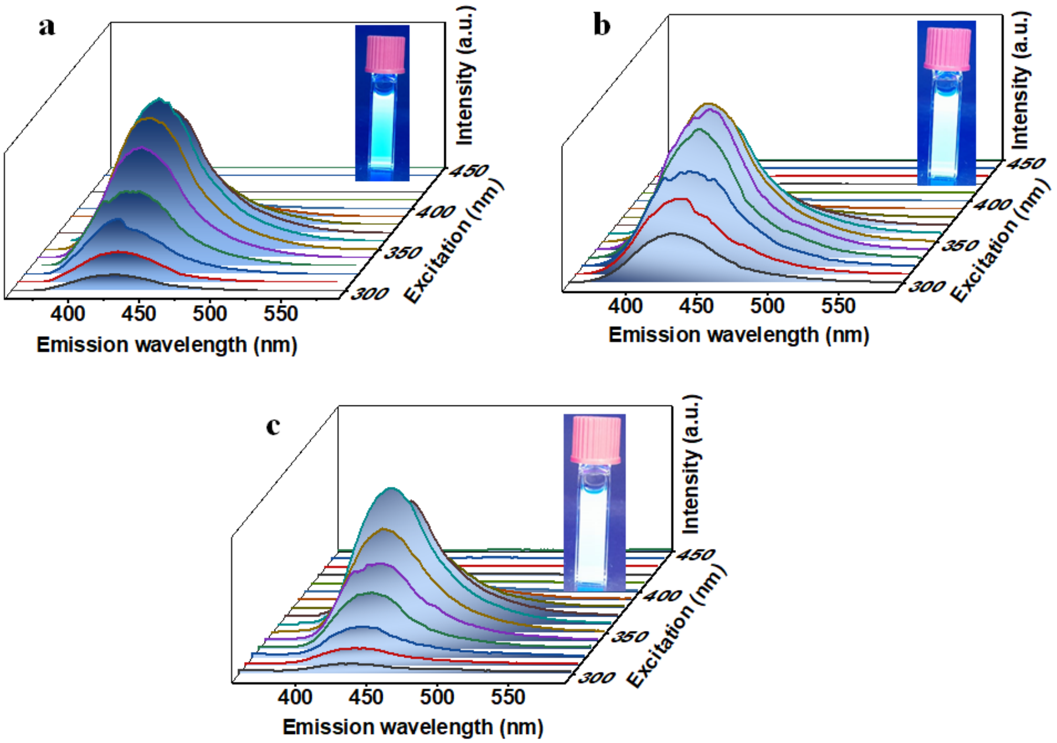

Figure S8. Excitation dependent emission spectra of (a) Cys CNDs, (b) Thr-CNDs and (c) Glu-CNDs. Insets show the photographs of the corresponding CND samples upon illumination using 360 nm UV lamp.

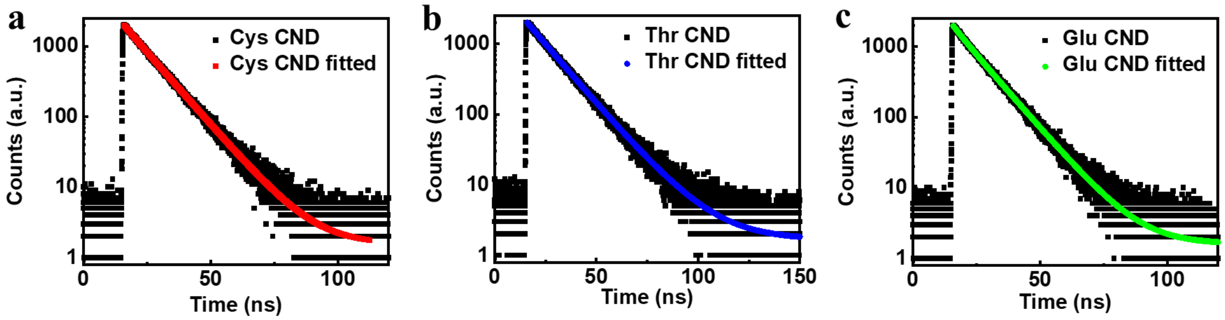

Figure S9. Lifetime decay curves of (a) Cys CNDs, (c) Thr CNDs, and (b) Glu CNDs.

Table S2. Summary of the fluorescence lifetime data

CNDs	τ (ns)	Rel.%
Cys-CNDs	10.6	98.7 %
	4.5	1.3 %
Thr-CNDs	12.5	89.1 %
	19	10.9 %
Glu-CNDs	10.5	96 %
	4.5	4%

Table S3. g_{abs} values of the chiral CNDs.

CNDs	g_{abs} for L CNDs	g_{abs} for D CNDs
Cys-CNDs	-4.5×10^{-4}	3.1×10^{-4}
Thr-CNDs	-1.0×10^{-4}	1.3×10^{-4}
Glu-CNDs	-2.6×10^{-4}	-

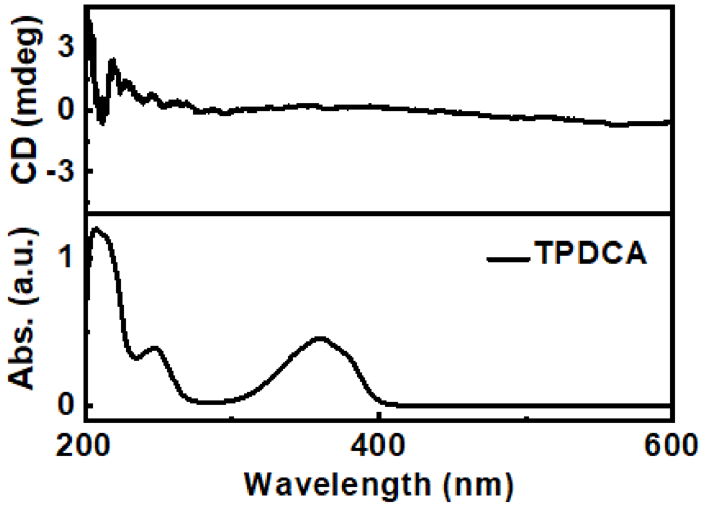

Figure S10. UV-vis (bottom) and CD (top) profiles of 5-oxo-3,5-dihydro-2H-thiazolo[3,2-a]pyridine-3,7-dicarboxylic acid (TPDCA).

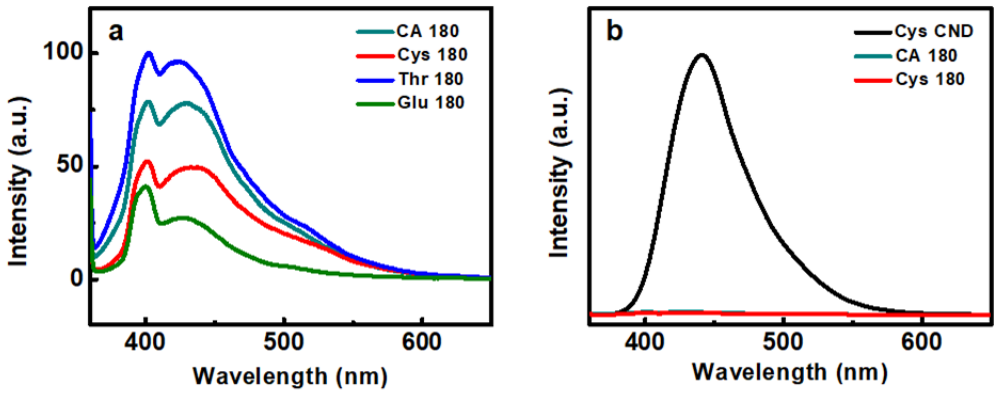

Figure S11. (a) Emission spectra of citric acid, cysteine, threonine, and glutathione samples after hydrothermal treatment at 180 oC for 1.5 h. (b) Emission spectra comparing the luminescence of Cys-CND (black trace) with its precursors, citric acid and cysteine, heated to 180oC for 1.5 h. *(Note: Sample obtained by the hydrothermal treatment of starting precursors exhibited negligible emission compared with the carbon nanodots).*

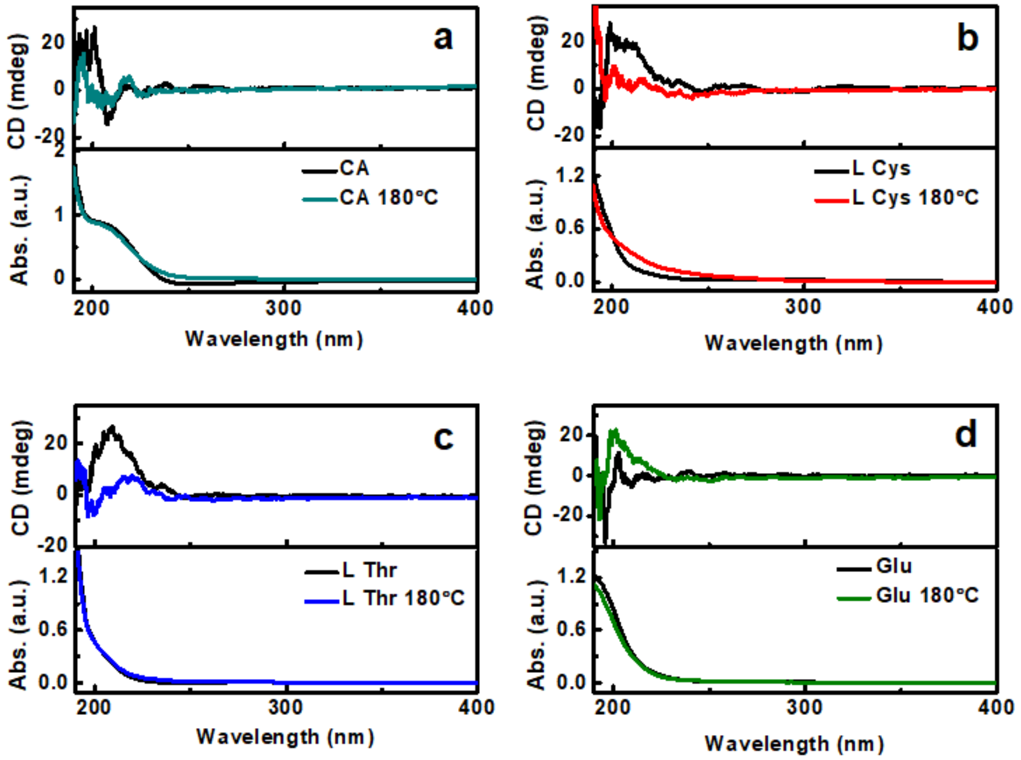

Figure S12. CD and the corresponding absorption spectra of (a) citric acid, (b) cysteine, (c) threonine (d) glutathione before and after heating at 180 °C.

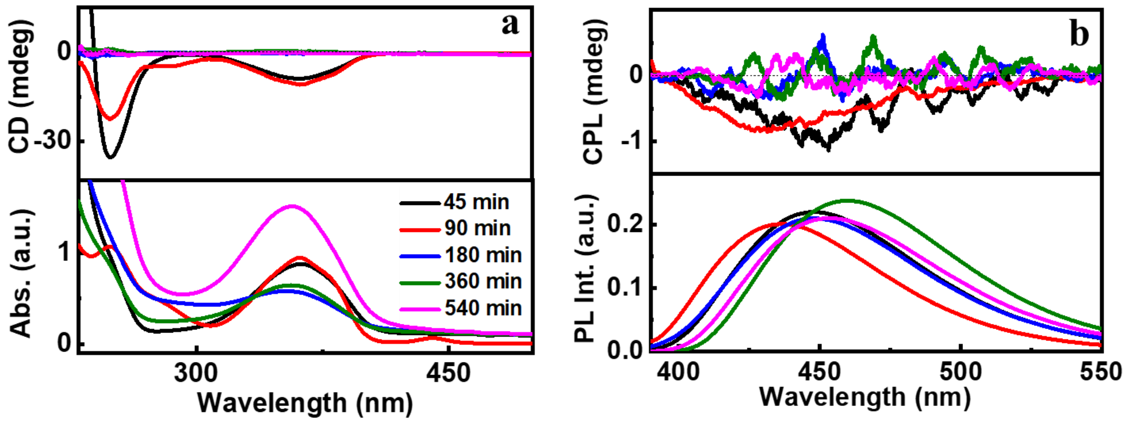

Figure S13. (a) CD (top) and the corresponding UV-vis spectra (bottom), and (b) CPL (top) and the corresponding fluorescence spectra (bottom) of Cys-CNDs synthesized at different reaction times (45, 90, 180, 360 and 540 min).

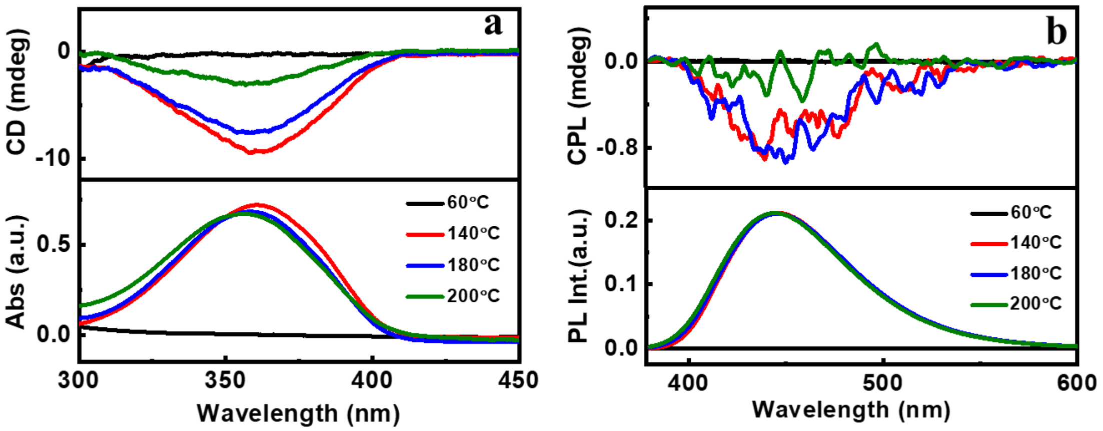

Figure S14. (a) CD (top) and the corresponding UV-vis spectra (bottom), and (b) CPL (top) and the corresponding fluorescence spectra (bottom) of Cys-CNDs synthesized at different reaction temperatures (60, 100, 140, 180 and 200 ºC).

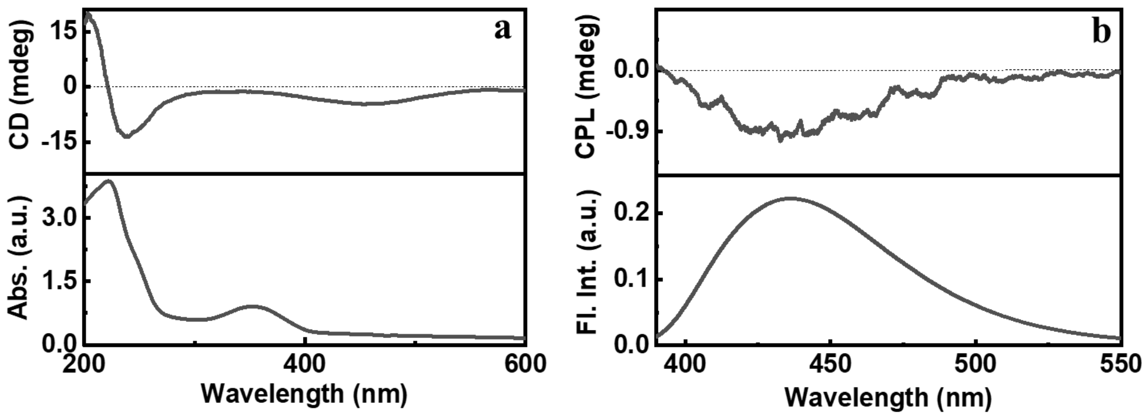

Figure S15. (a) CD (top) and the corresponding UV-vis spectra (bottom), and (b) CPL (top) and the corresponding fluorescence spectra (bottom) of Glu-CNDs synthesized using L-glutathione.

Table S4. g_{lum} values from chiral CNDs in solution state

Compound	g_{lum} with L isomer	g_{lum} with D isomer
Cys-CNDs	-7 x 10^{-4}	8 x 10^{-4}
Thr-CNDs	-2.16 x 10^{-4}	2.6 x 10^{-4}
Glu-CNDs	-3 x 10^{-4}	-

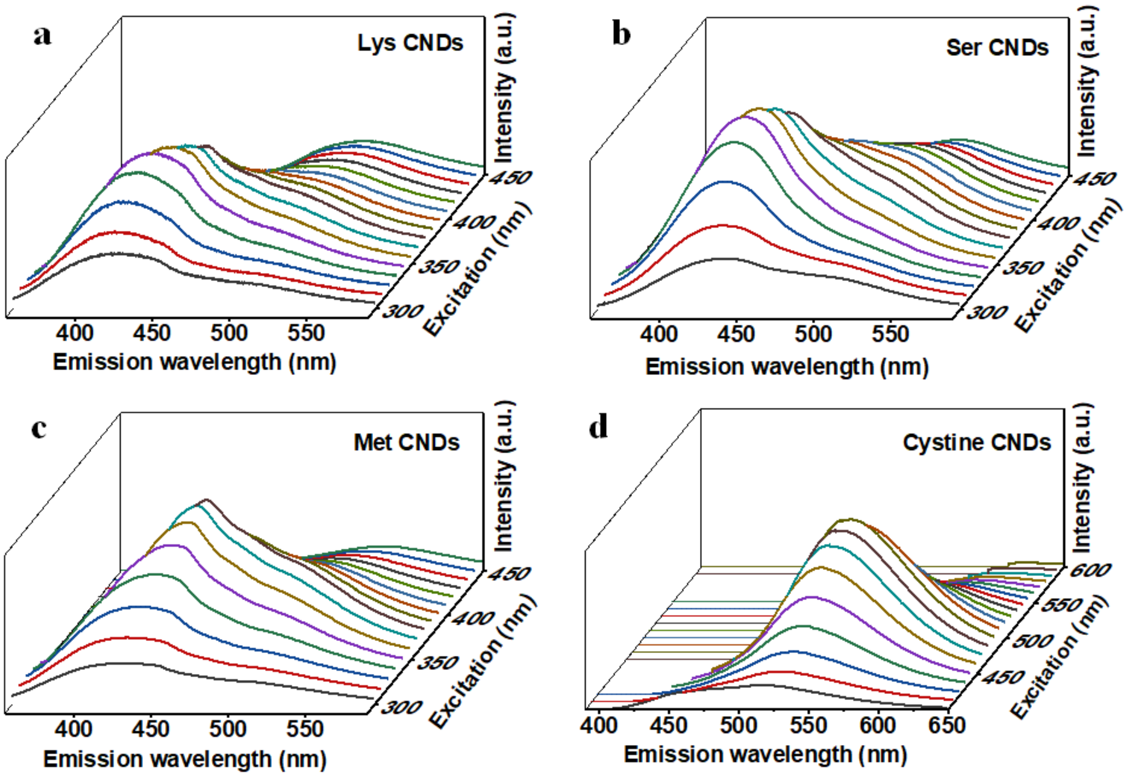

Figure S16. Excitation-dependent emission spectra of CNDs synthesized using different amino acids; (a) lysine, (b) serine, (c) methionine and (d) cystine.

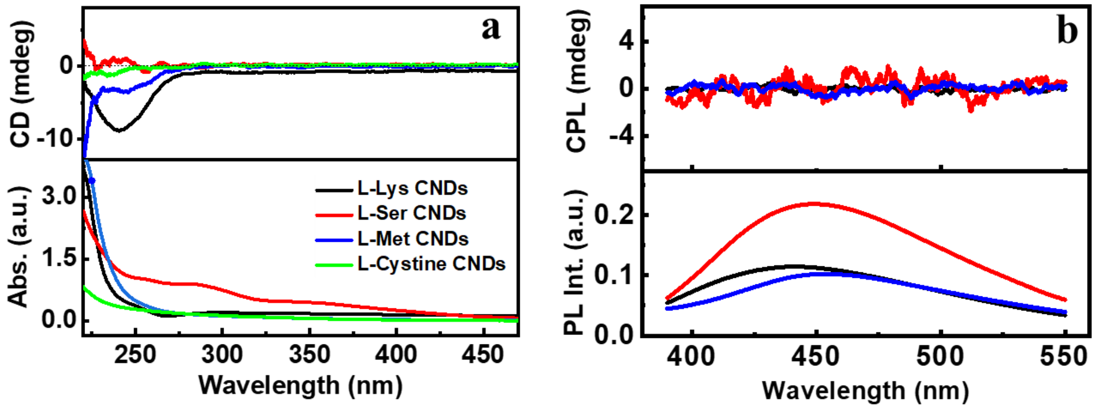

Figure S17. (a) CD (top) and the corresponding UV-vis spectra (bottom), and (b) CPL (top) and the corresponding fluorescence spectra (bottom) of CNDs synthesized using lysine, serine, methionine and cystine.

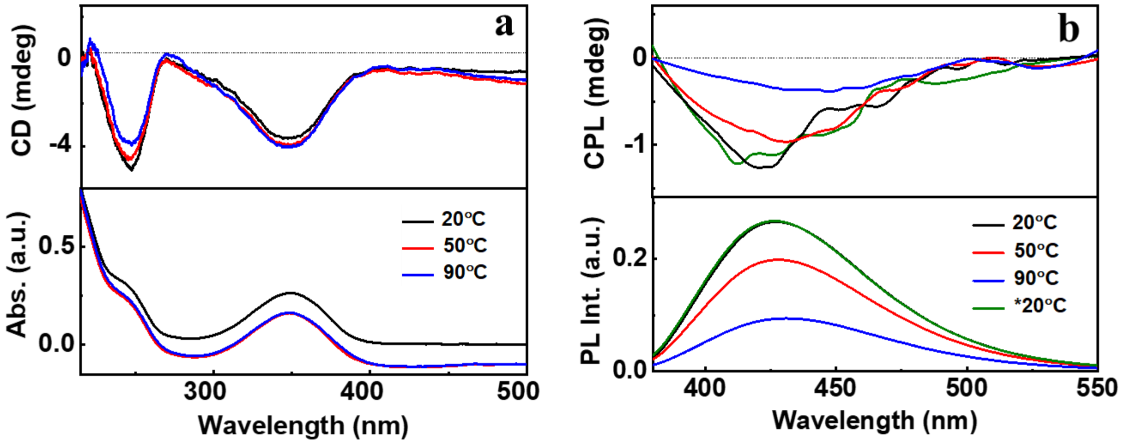

Figure S18. (a) CD (top) and the corresponding UV-vis spectra (bottom), and (b) CPL (top) and the corresponding fluorescence spectra (bottom) of Cys-CNDs on heating and cooling. (*20 ºC indicates cooling the sample back to 20 ºC after heating till 90 ºC).

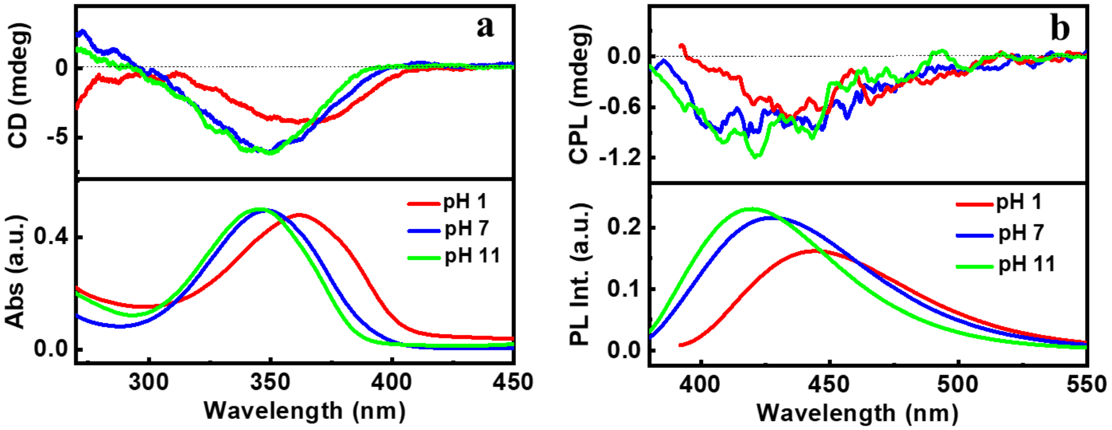

Figure S19. (a) CD (top) and the corresponding UV-vis spectra (bottom), and (b) CPL (top) and the corresponding fluorescence spectra (bottom) of Cys-CNDs at different pH.

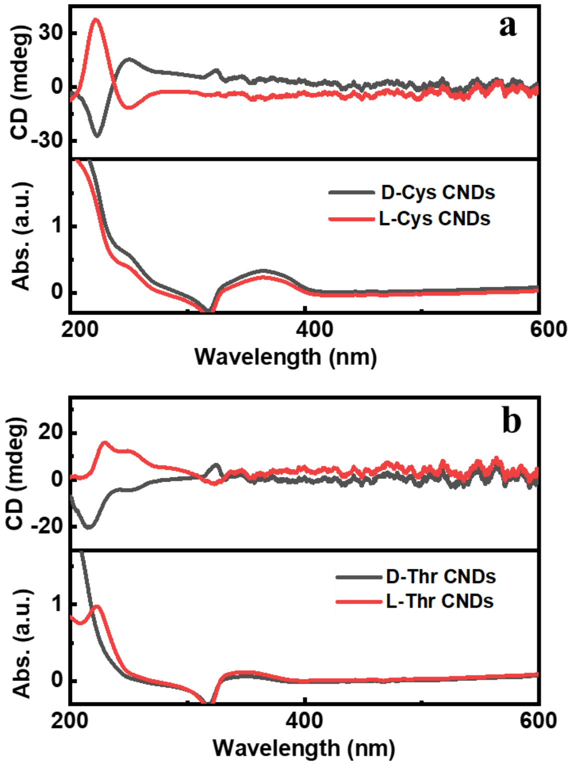

Figure S20. (a) CD (top) and the corresponding UV-vis spectra (bottom) of polymeric films of (a) Cys-CNDs and (b) Thr-CNDs in solid state.

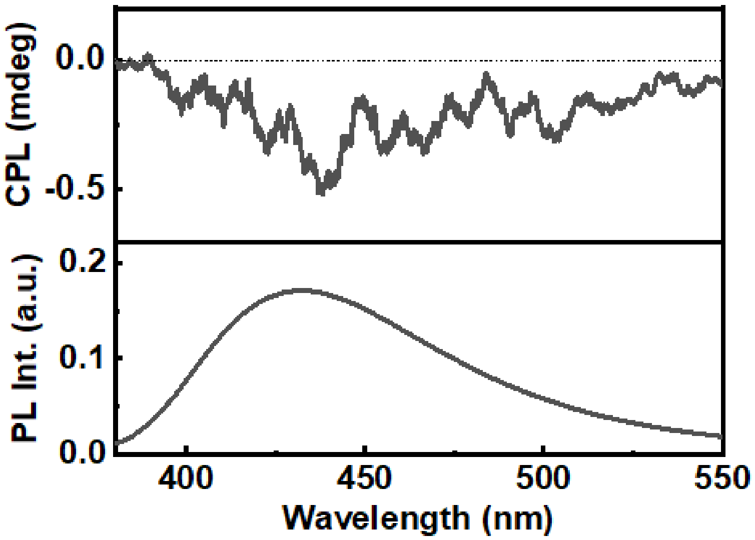

Figure S21. CPL (top) and fluorescence spectra (bottom) of polymeric films of Glu-CNDs.

Table S5: g_{lum} values from chiral CNDs in solid state

CNDs	g_{lum} with L isomer	g_{lum} with D isomer
Cys-CNDs	-3.9×10^{-4}	5.9×10^{-4}
Thr-CNDs	-9×10^{-4}	6×10^{-4}
Glu-CNDs	-2×10^{-4}	-

References

1. S. Cailotto, R. Mazzaro, F. Enrichi, A. Vomiero, M. Selva, E. Cattaruzza, D. Cristofori, E. Amadio, A Perosa, Design of Carbon Dots for Metal-free Photoredox Catalysis, *ACS Appl. Mater. Interfaces* 2018, **10**, 40560−40567.

2. V. Strauss, A. Kahnt, E. M. Zolnhofer, K. Meyer, H. Maid, C. Placht, W. Bauer, T. J. Nacken, W. Peukert, S. H. Etschel, M. Halik, D. M. Guldi, Assigning Electronic States in Carbon Nanodots, *Adv. Funct. Mater.*, 2016, **26**, 7975−7985.
